# Silver Nanoparticles and *Glycyrrhiza glabra* (Licorice) Root Extract as Modifying Agents of Hydrogels Designed as Innovative Dressings

**DOI:** 10.3390/ijms24010217

**Published:** 2022-12-22

**Authors:** Magdalena Kędzierska, Magdalena Bańkosz, Anna Drabczyk, Sonia Kudłacik-Kramarczyk, Mateusz Jamroży, Piotr Potemski

**Affiliations:** 1 Department of Chemotherapy, Medical University of Lodz, Copernicus Memorial Hospital of Lodz, 93-513 Lodz, Poland; 2 Department of Materials Engineering, Faculty of Materials Engineering and Physics, Cracow University of Technology, 37 Jana Pawła II Av., 31-864 Krakow, Poland; 3 Faculty of Materials Engineering and Physics, Cracow University of Technology, 37 Jana Pawła II Av., 31-864 Krakow, Poland

**Keywords:** hydrogel dressings, licorice root extract, silver nanoparticles, wettability, cytotoxicity, tensile strength, sorption ability

## Abstract

The interest in the application of plant extracts as modifiers of polymers intended for biomedical purposes is constantly increasing. The therapeutical properties of the licorice root, including its anti-inflammatory and antibacterial activity, make this plant particularly promising. The same applies to silver nanoparticles showing antibacterial properties. Thus the main purpose of the research was to design hydrogel dressings containing both licorice root extract and nanosilver so as to obtain a system promoting wound regeneration processes by preventing infection and inflammation within the wound. The first step included the preparation of the plant extract via the solid-liquid extraction using the Soxhlet extractor and the synthesis of silver nanoparticles by the chemical reduction of silver ions using a sodium borohydride as a reducing agent. Subsequently, hydrogels were synthesized via photopolymerization and subjected to studies aiming at characterizing their sorption properties, surface morphology via scanning electron microscopy, and their impact on simulated physiological liquids supported by defining these liquids’ influence on hydrogels’ structures by FT-IR spectroscopy. Next, the tensile strength of hydrogels and their percentage elongation were determined. Performed studies also allowed for determining the hydrogels’ wettability and free surface energies. Finally, the cytotoxicity of hydrogels towards L929 murine fibroblasts via the MTT reduction assay was also verified. It was demonstrated that developed materials showed stability in simulated physiological liquids. Moreover, hydrogels were characterized by high elasticity (percentage elongation within the range of 24–29%), and their surfaces were hydrophilic (wetting angles below 90°). Hydrogels containing both licorice extract and nanosilver showed smooth and homogeneous surfaces. Importantly, cytotoxic properties towards L929 murine fibroblasts were excluded; thus, developed materials seem to have great potential for application as innovative dressings.

## 1. Introduction

*Glycyrrhiza glabra* (licorice) root is widely applied as a sweetener and flavoring agent in candies, sweets, and other food products. The sweet taste of licorice root results from the presence of glycyrrhizin [1,2]. However, despite its use in the food industry, this plant is also applied as a medicinal herb [3]. Licorice root has been used in Chinese medicine for over 1000 years. Its pharmacological properties result from the fact that it consists of a huge amount of active chemical compounds, including isoflavonoids, chalcones, amino acids, lignins, amines, gums, and volatile oils. It was demonstrated that the composition of licorice root contains over 20 triterpenoids and 300 flavonoids [4,5]. Such an extensive chemical composition makes this plant an extremely interesting research material in terms of the possibilities of its pharmaceutical applications. Biologically active licorice compounds are used in many diseases due to their anti-inflammatory, antibacterial, and antiviral properties [6,7,8]. Furthermore, licorice also shows anti-carcinogenic and neuroprotective activity (it may prevent or delay the degradation of neurons) [9]. It has also been proven that licorice may be applied as an effective therapeutic agent in the treatment of diabetes [10]. As has been demonstrated by Yang et al., licorice extracts show excellent anti-diabetic activity both in vitro and in vivo. They affect the mechanisms of insulin receptor site sensitivity, increase the use of glucose in various tissues and organs, and correct metabolic disorders improving microcirculation in the body simultaneously [11]. Subsequently, it has also been reported that glabrydin occurring in the licorice root may be an alternative therapeutic agent in thrombogenic disorders due to the impact of this compound on the prevention of platelet aggregation [12]. *Glycyrrhiza* L. root may also be successfully used in the treatment of infectious hepatitis and bronchitis [13]. Feng Yeh et al. proved the effective activity of licorice extract against HRSV infection on airway epithelial cells. Bioactive compounds present in this plant extract prevented virus transfer and internalization. Moreover, it has been demonstrated that under the influence of *Glycyrrhiza* L., mucosal cells are stimulated to release IFN-β, counteracting the same viral infection [14]. It has also been stated that the flavonoids extracted from licorice root reduced the inflammation of acute pneumonia induced in vivo in mice by lipopolysaccharide [15]. Studies on the application of licorice extracts in the treatment of respiratory diseases, including COVID-19, were also presented by Gomaa et al. [16] and li Ng et al. [17]. Additionally, the possibility of the application of licorice extracts in the treatment of alcoholic liver injury was described in [18]. Furthermore, the attention of scientists was focused not only on the therapeutic effect of the bioactive compounds included in licorice but also on their ability to enhance the action of the other medicinal compounds by forming supermolecular complexes with them [19]. Due to its antioxidant, anti-inflammatory, and antibacterial properties, many studies have been on the use of licorice in the treatment of inflammation accompanying certain skin diseases [20]. It has been shown that the preparations based on licorice extract may be successfully applied in the treatment of erythema [21], vitiligo [22,23], atopic dermatitis [24,25], as well as baldness [26].

The wide spectrum of the activity of the compounds included in the licorice root corresponds to the great application potential of this plant. Thus considering its properties desirable in terms of skin inflammation treatment, the main purpose of the presented research was to develop hydrogel dressing material modified with licorice root extract. Many commercially available preparations occur in the form of a gel of an ointment wherein such forms may cause some of the active substance to rub into clothing; thus, the introduction of such a substance into the hydrogel dressing may prevent this phenomenon. In this work, the results of the research aimed at developing hydrogel dressing materials containing bioactive substances extracted from licorice root. Furthermore, developed materials were additionally incorporated with silver nanoparticles showing antibacterial properties. The main hypothesis of the research was to develop a multifunctional dressing material that could simultaneously fulfill protective functions, absorb wound exudate, and show anti-inflammatory and antibacterial properties due to the presence of the modifiers: licorice root extract and nanosilver. To our knowledge, such a combination is innovative and has not yet been presented. The base forming the hydrogel matrix consisted of natural polymers such as gelatin and chitosan, demonstrating high biocompatibility. In the first step of the research, the synthesis of silver nanoparticles was performed, while for this purpose, the chemical reduction was employed. Next, licorice extract was prepared via the Soxhlet extraction. Both these materials were subsequently used as modifiers of chitosan/gelatin-based hydrogel dressings. The hydrogels were obtained by means of UV radiation and next subjected to the detailed physicochemical characteristic. In the course of the research, sorption properties of hydrogels important in terms of potential sorption of the wound exudate were determined. Next, incubation of hydrogels in simulated physiological liquids supported by determining their impact on hydrogels’ structure via FT-IR spectroscopy was also performed. Hydrogels’ surface morphology, wettability as well as mechanical properties, including the tensile strength and the percentage elongation, were also characterized. Finally, the cytotoxicity of hydrogels towards L929 murine fibroblasts via the MTT reduction assay was also verified.

## 2. Results and Discussion

### 2.1. Characteristic of Nanosilver Suspension

#### 2.1.1. The Particle Size Analysis via DLS Technique

**Figure 1:**
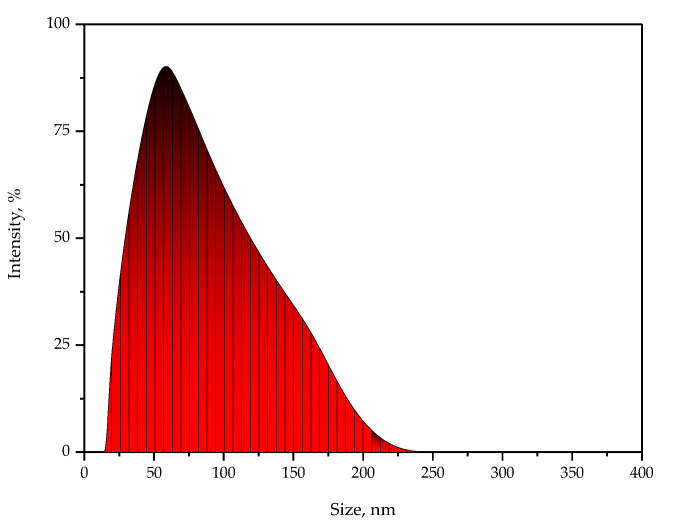
The particle size distribution in obtained silver nanoparticles suspension.

Below in Figure 1, the results of the DLS analysis are presented.

Based on the performed analysis, it may be reported that in the tested suspension, the particles with sizes within the range of 20–200 nm occurred, wherein the most common population of the particles showed the size with an average hydrodynamic diameter within the range of 40–80 nm. Thus the results of the DLS analysis clearly indicated the presence of nanoparticles in the suspension.

#### 2.1.2. Characterization of Optical Properties of Nanosilver Suspension

**Figure 2:**
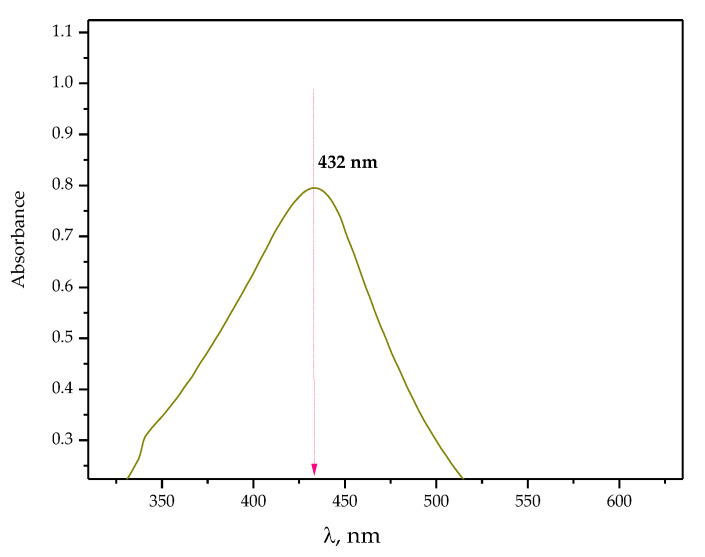
UV–Vis spectrum of the particle suspension obtained as a result of the chemical reduction of silver ions.

UV–Vis spectrum of the suspension obtained as a result of the chemical reduction of silver ions is presented below in Figure 2. The analysis allowed us to verify whether silver nanoparticles were obtained as a result of the method applied.

**Figure 3:**
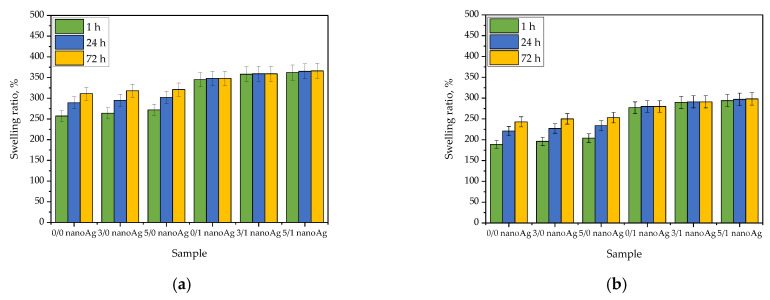

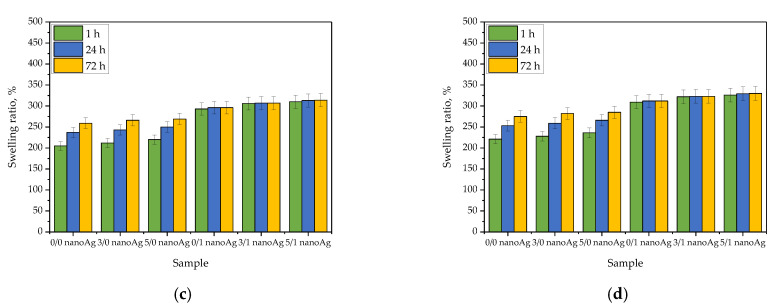
Results of investigations on sorption properties of hydrogels in distilled water (**a**), SBF (**b**), Ringer liquid (**c**), and artificial saliva (**d**) (n = 3, n—number of repetitions).

In order to confirm the presence of the silver nanoparticles, the suspension obtained as a result of the procedure described in Section 3.2. of the paper was subjected to UV–Vis spectroscopy. Noble metal nanoparticles, including silver ones, are characterized by specific optical properties as well as the ability to absorb radiation within the range of visible and ultraviolet. Thus, UV–Vis spectroscopy is one of the most frequently used methods aimed at confirming the presence of silver nanoparticles [27,28]. On the UV–Vis spectrum visible in Figure 3, the absorption band with a maximum at a wavelength of approximately 432 nm, typical exactly for silver nanoparticles, was observed. The absorption band at a similar wavelength demonstrating the presence of nanosilver was also presented by Agustina et al. [29] and Alim-Al-Razy et al. [30]. This, in turn, indicates the consistency of the results of the performed analysis with previously reported studies.

### 2.2. Results of Hydrogels’ Swelling Ability Measurements

Swelling ratios of tested hydrogels are presented below in Figure 3, wherein the results are compiled separately for each liquid. The study was conducted in triplicates, and the results are shown as average values with corresponding standard deviations (SD, presented as error bars).

Studies on the swelling properties of the materials are aimed at determining the ability of the material to interact with aqueous solutions, which means its ability to their absorption. Such ability may be expressed as the swelling ratio (with unit %) after the appropriate calculation, including the mass of a dry hydrogel and a mass of hydrogel after swelling at a specific time. So based on such calculations for performed experiments, it may be concluded that tested hydrogels showed a sorption capability of about 200–300%. Importantly, the highest values of this parameter were reported for hydrogels swelling in distilled water, wherein the lowest ones were in the case of materials tested in SBF. High values of swelling ratio in selected liquid mean that the samples absorbed this liquid to the greatest extent. Distilled water, contrary to the rest of the tested media, does not contain any ions which could negatively affect the sorption process of the tested material. For example, divalent ions such as calcium ions may interact with the polymer network, resulting in an increase in the crosslinking density of polymer chains. This, in turn, may result in a decrease in the swelling properties of such polymer. This dependence is clearly visible in the case of the results presented in Figure 3b–d, where the calculated swelling ratios are lower than the ones presented in Figure 3a.

Results of performed studies also allow for determining the impact of the modifiers—licorice root extract and nanosilver suspension—on the sorption properties of hydrogels. Thus, referring to the first mentioned modifier, it may be reported that the presence of the plant extract in the hydrogel matrix did not affect the swelling properties of tested materials. Due to the fact that hydrogels showing high swelling properties are considered beneficial in terms of their biomedical application as dressings, the lack of the negative impact of the licorice root extract on this property is determined as a positive aspect. Thus it may be concluded that the mentioned extract does not affect the swelling ability and, importantly, does not reduce this ability compared to the swelling properties of hydrogel without this additive. Another situation was observed in the case of the modification of hydrogels with nanosilver. A clear increase in the swelling ratios of hydrogels containing this modifier was reported. Probably this resulted from the interactions between the aqueous suspension of silver nanoparticles and the hydroxyl groups included in the medium in which the swelling samples were placed. These interactions cause the attraction of the water molecules and thus contribute to the higher values of the swelling ratios observed on the y-axis. From the application viewpoint, such a phenomenon is most desirable. Nonetheless, it should be emphasized that the appropriate selection of the components of the hydrogel matrix allows for controlling the sorption properties of the tested materials.

### 2.3. Results of Incubation Studies

**Figure 4:**
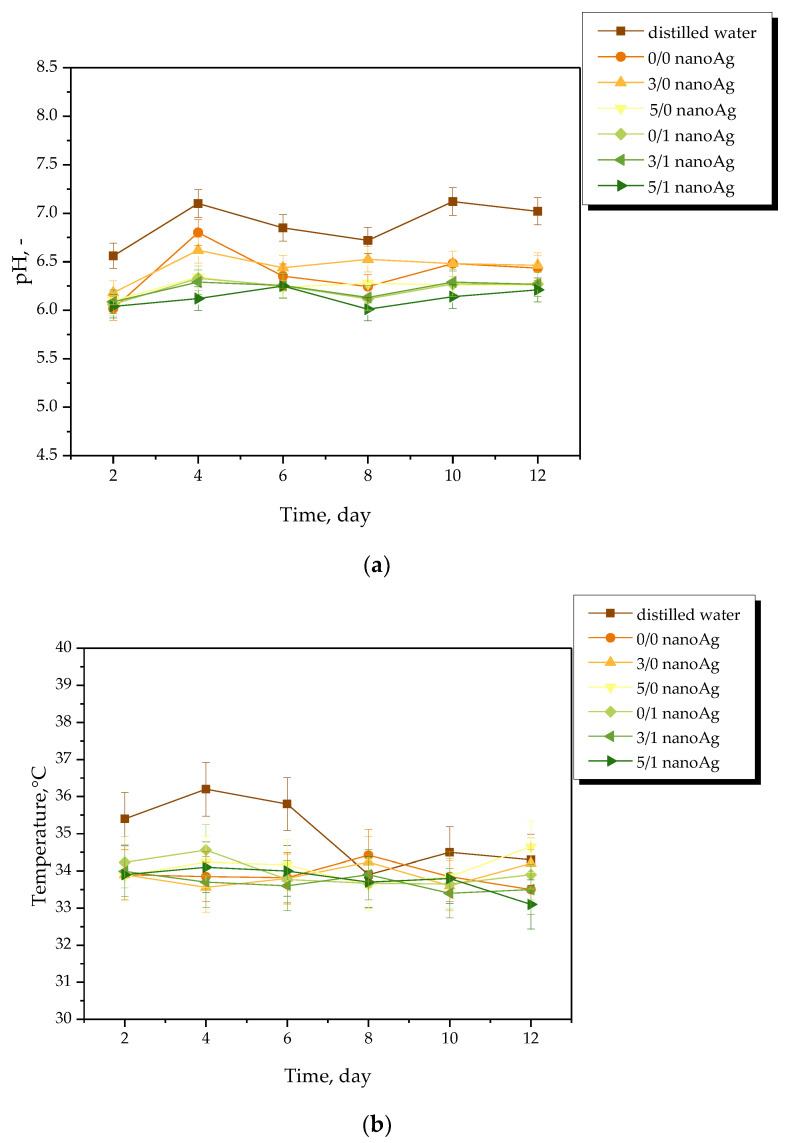
pH (**a**) and temperature (**b**) measurements of distilled water during hydrogels’ incubation (n = 3, n—number of repetitions).

**Figure 5:**
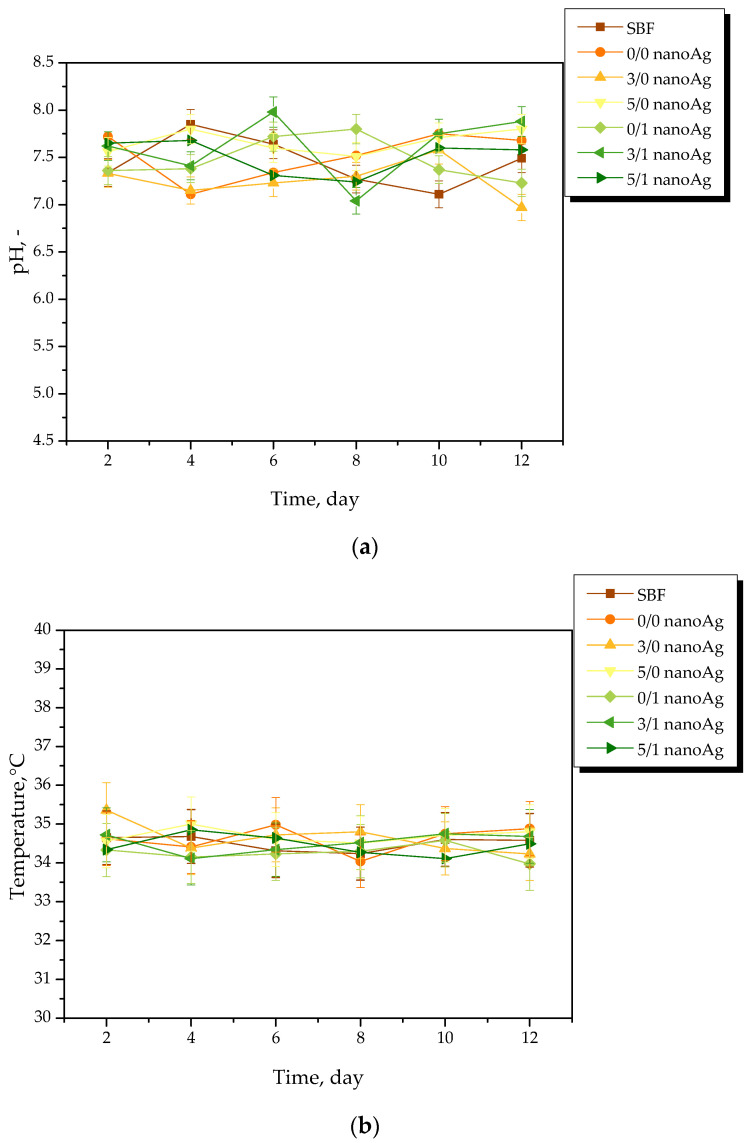
pH (**a**) and temperature (**b**) measurements of SBF during hydrogels’ incubation (n = 3, n—number of repetitions).

**Figure 6:**
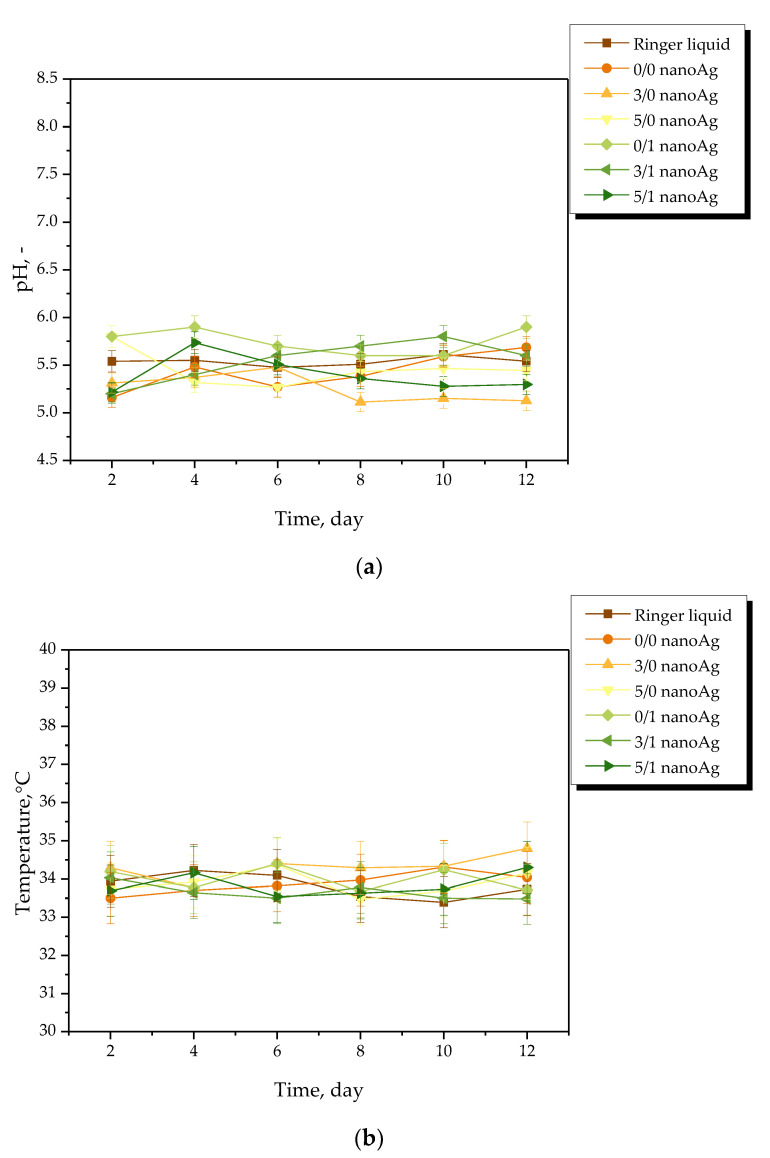
pH (**a**) and temperature (**b**) measurements of Ringer liquid during hydrogels’ incubation (n = 3, n—number of repetitions).

**Figure 7:**
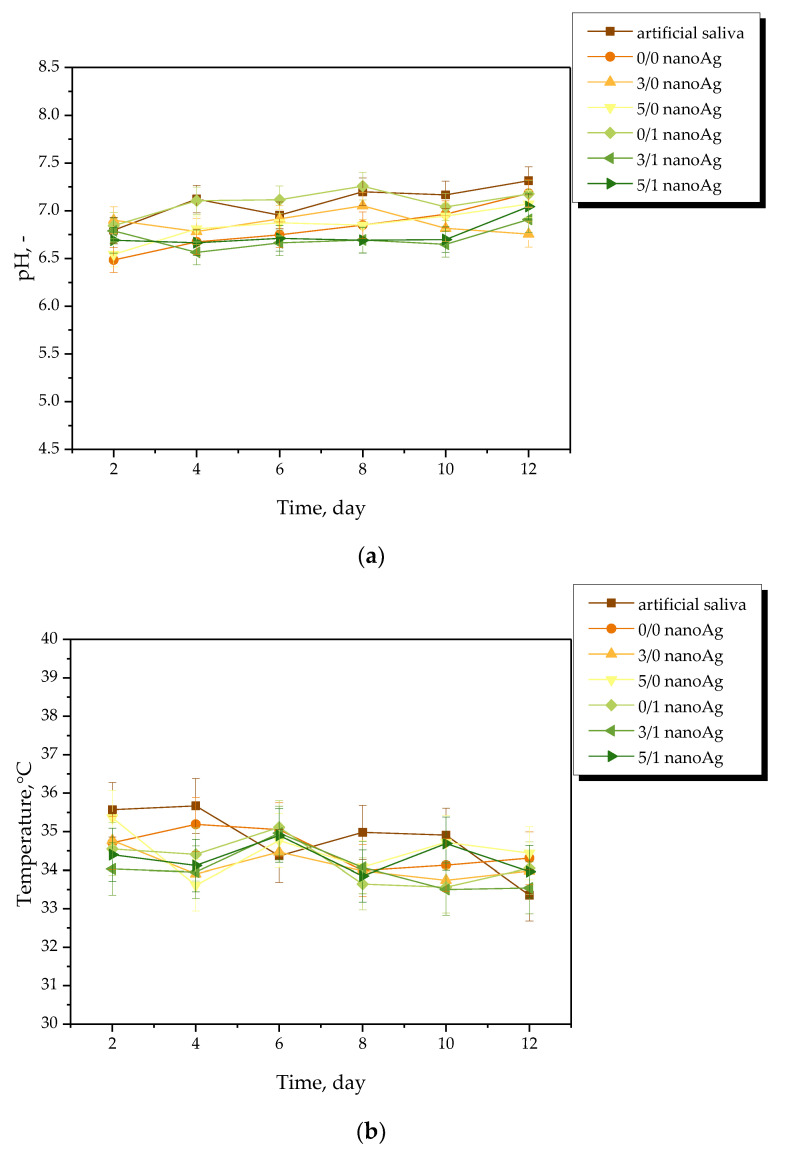
pH (**a**) and temperature (**b**) measurements of artificial saliva during hydrogels’ incubation (n = 3, n—number of repetitions).

Results of the measurements of the pH and the temperature of incubation media in the presence of the hydrogel samples are presented in Figure 4, Figure 5, Figure 6 and Figure 7. The study was performed in triplicates wherein the results are given as average values with corresponding standard deviations (SD, shown as error bars).

The occurrence of rapid changes in pH may indicate the degradation of hydrogel materials or the release of unreacted reagents, such as a crosslinking agent or photoinitiator from the matrix, which is an undesirable phenomenon. In the case of all tested materials, such rapid and significant changes in the values of tested parameters have not been observed. This, in turn, indicates the stability of the hydrogels in tested environments. However, some slight changes of various natures—i.e., decreases or increases—in measured pH values of the incubation media were noticed. Nonetheless, these changes were very slight, i.e., by a maximum of one pH unit. The hydrogel swells under the influence of the incubation medium; this phenomenon has been described in more detail in Section 2.2. As a result of liquid sorption, the loosening of the polymer network may take place. This, in turn, may lead to the occurrence of various interactions between the components of the polymer and the incubation medium. As a result, numerous compounds (both acidic and alkaline in nature) included in the licorice root extract acting as a modifier of the hydrogel may release from the polymer matrix. Finally, slight changes in pH of the incubation media in which such a release takes place may be observed. Alternating changes in pH values (i.e., their slight decreases or increases) may be caused by constant interactions of the incubation material with the liquid. Such a phenomenon indicating the gradual release of active compounds present in licorice root extract is beneficial due to the fact that it gives information on the development of the materials with active substance delivery function.

### 2.4. The Impact of the Incubation Studies on Hydrogels’ Chemical Structure Verified via FT-IR Spectroscopy

**Figure 8:**
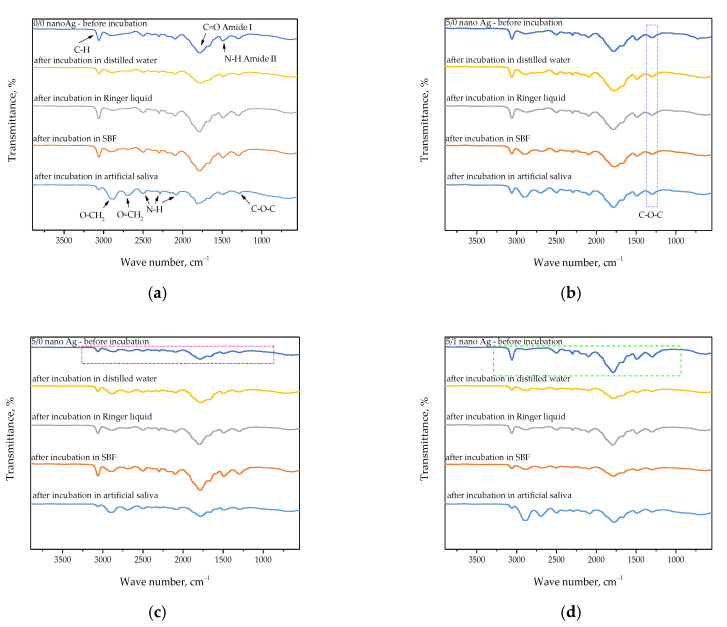
FT-IR spectra showing the impact of the incubation on the structure of sample 0/0 nanoAg (**a**), 5/0 nanoAg (**b**), 0/1 nanoAg (**c**), and 5/1 nanoAg (**d**).

FT-IR spectra of hydrogels are presented in Figure 8. The spectra were compiled in such a way as to compare the structure of particular samples before and after the incubation in tested media.

The performed spectroscopic analysis allowed for verifying the presence of characteristic groups for compounds included in developed polymer matrices. The study was performed both for samples before and after the incubation in simulated physiological liquids. On the one hand, the disappearance of characteristic absorption bands may suggest the degradation of the tested material. On the other hand, it may be reported that such a degradation did not occur because this process would be reflected in significant pH changes of incubation media, and such ones have not been observed. Thus such disappearance or decrease in the intensity of some absorption bands may also indicate the release of the modifiers—i.e., licorice root extract or silver nanoparticles—which, located within the polymer network, may obscure some characteristic groups (as a result, the signal deriving from them is limited).

In Figure 8a, it is possible to observe the absorption bands characteristic of the structure of two polymers used for the synthesis of hydrogel matrix, i.e., chitosan and gelatin [31,32,33]. Additionally, on FT-IR spectra of the sample after incubation in artificial saliva, an occurrence of more absorption bands characteristic for these polymers—invisible on the spectrum of the material prior to incubation—was observed (Figure 8a). This may suggest that the polymer chains in the tested material after the drying process were arranged in a way that allowed the disclosure and detection of more groups characteristic for components forming the polymer matrix. Such a dependence was also observed for other tested materials, i.e., in Figure 8b–d). This probably results from the chemical composition of the artificial saliva and the interactions occurring between the functional groups of the polymers and ions included in this incubation liquid.

An interesting dependence was also observed in the case of the hydrogel modified with licorice root extract. In Figure 8b), an increase in the intensity of the absorption band characteristic for the –C–O–C– group deriving from polysaccharides included in this extract may be observed (this has been marked via the blue frame in Figure 8b). Thus, this demonstrates that the presence of the additive in the form of the plant extract is indicated by the increase in the intensity of the selected absorption band in the FT-IR spectra.

Another equally interesting dependence observable during the performed study may be noticed in the FT-IR spectra of hydrogels incorporated with silver nanoparticles. In Figure 8c,d, the spectra of materials before incubation may be observed (the absorption bands have been marked via the frames—pink one in Figure 8c and green one in Figure 8d), wherein in the case of the material without the licorice root extract (Figure 8c), the spectrum is very blurry and differs significantly in the intensity from the spectrum visible in Figure 8d. In the case of the hydrogel with nanosilver, these nanoparticles are probably located between the polymer chains (in free spaces between them), which makes it difficult to identify groups deriving from polymers. However, a release of nanosilver or its elution probably takes place as a result of the incubation, which in turn results in exposing the characteristic groups giving the same appropriate absorption bands on FT-IR spectra. In the case of the hydrogels modified both with licorice root extract and nanosilver, such a phenomenon was not observed. This is probably caused by the different placement of metallic nanoparticles within the polymer structure. Additionally, some absorption bands derive also from the polysaccharides included in the plant extract.

### 2.5. Results of SEM Imaging of Hydrogels

**Figure 9:**
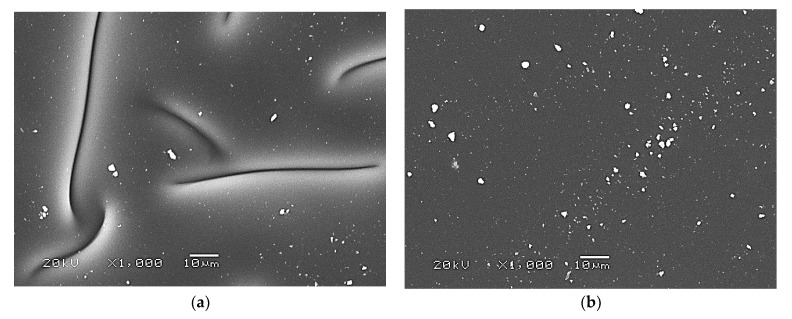

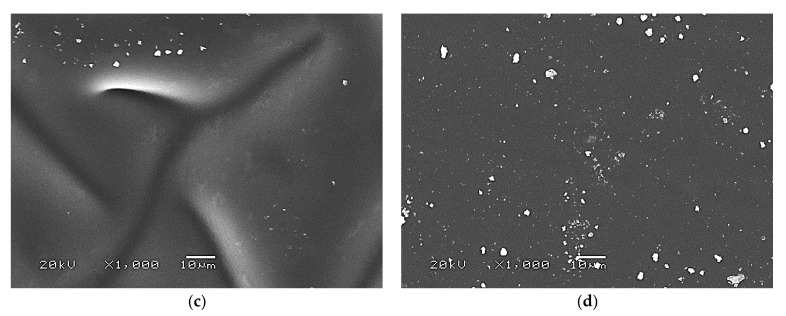
SEM images of hydrogel materials: sample: 0/0 nanoAg (**a**); 5/0 nanoAg (**b**); 0/1 nanoAg (**c**); 5/1 nanoAg (**d**).

The surface morphology of hydrogels was characterized by means of scanning electron microscopy. Obtained SEM images are presented in Figure 9.

Based on the presented SEM images, it may be reported that the highest impact on the surface morphology of the hydrogels has the presence of the plant extract. The samples without this additive (whose images are presented in Figure 9a,b) are characterized by heterogeneous and undulating surfaces. In the case of hydrogels modified with the mentioned extract, their surface is homogeneous and smooth (Figure 9b,d). The plant extract introduced into the polymer matrix probably fills the cavities on the polymer surface, thus making it smoother. On the other hand, any significant impact of nanosilver on the developed materials’ surface morphology was not observed.

### 2.6. Wettability of Hydrogels Supported by Determining Their Surface Free Energy

Next, in order to verify the hydrophilicity or hydrophobicity of the surfaces of developed hydrogels, their wetting angles were determined. The results of performed investigations supported by the images showing the first contact of the liquid of distilled water with the hydrogel sample are presented in Table 1, wherein the results of the statistical analysis are shown in Table 2.

Based on the performed analysis, it was reported that as the content of the modifiers in the polymer matrices increased, the values of their wetting angles decreased. In the case of the unmodified hydrogel sample, its wetting angle was 42°, wherein the value of this parameter determined for the sample containing both these additives (i.e., 5 mL of the plant extract and 1 mL of nanosilver suspension) was 26°. Hydrophilic surfaces are defined as the ones for which the value of their wetting angle is lower than 90° [34]. Thus it may be concluded that all analyzed materials showed a hydrophilic surface wherein the larger the amount of the modifiers, the higher hydrophilicity. Due to the presence of the chemical compounds included in licorice root extract in the modified materials, interactions in the form of hydrogen bonds between functional groups from these compounds and the water molecules may occur, thus increasing the wettability of such materials’ surface. Moreover, in the case of the presence of nanosilver, which was introduced into the polymer matrix in the form of an aqueous suspension, such interactions between modified hydrogels and the drop of liquid may occur, which also translates into the decrease of the wetting angle. The surface wettability is strictly correlated with the value of its surface free energy. Along with the decreasing values of the contact angle, the increase in the value of the surface free energy, which may be defined as a measure of the attractive force of the tested substrate, is observed. The highest value of the total surface free energy was reported in the case of the samples characterized by the lowest wetting angle and thus the most hydrophilic surface, i.e., sample 5/1 nanoAg (modified with the highest amounts of licorice root extract and nanosilver).

The material’s surface, its roughness, wettability, topography, as well as surface free energy constitute the very important parameters characterizing the biomaterial. The initial cell adhesion, which depends to a large extent on the aforementioned parameters, is of key importance for further cell proliferation and their regenerative processes [35]. As it was reported by Majhy et al., a moderate surface free energy of 70 mJ/m^2^ is the most favorable for effective cell adhesion, growth, and proliferation [36]. This is consistent with other works where it was demonstrated that cells show better adhesion to hydrophilic surfaces [37]. Considering the obtained results, it may be concluded that developed materials, due to the surfaces’ hydrophilic nature and the high surface free energy values, show the desired features in terms of supporting regeneration processes.

### 2.7. Results of Mechanical Investigations including Determining the Hydrogels’ Tensile Strength and Percentage Elongation

**Figure 10:**
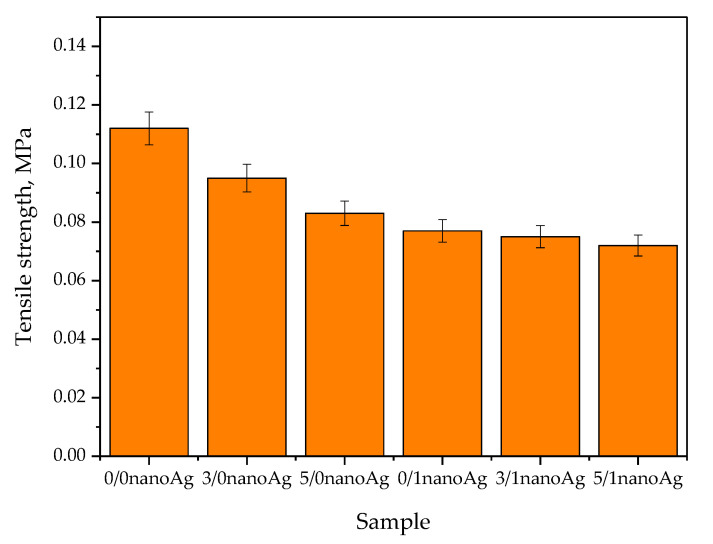
Results of tensile strength measurements of hydrogels (number of repetitions n = 3).

**Figure 11:**
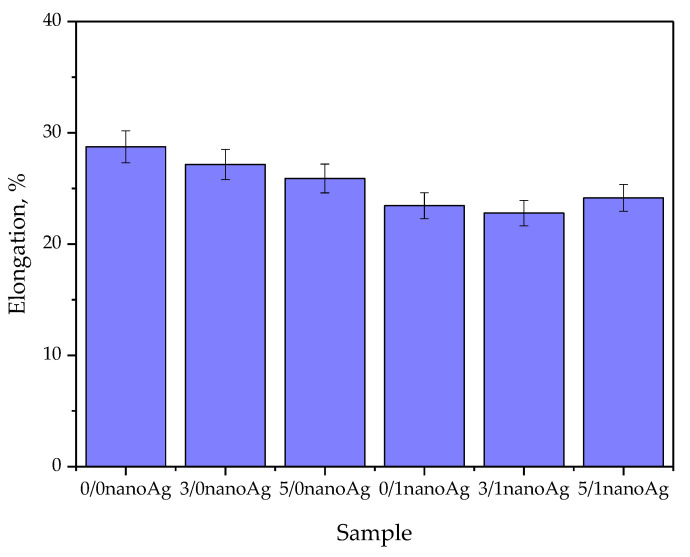
Results of percentage elongation measurements of hydrogels (number of repetitions n = 3).

Results of studies on the tensile strength of hydrogels are presented in Figure 10, the values of their percentage elongation are shown in Figure 11, and the results of the statistical analysis are shown in Table 3 and Table 4.

Materials, which are considered for applications for biomedical purposes, should meet a number of requirements. Apart from biocompatibility and relatively simple and quick synthesis methodology, their mechanical properties are extremely important [38]. One of the most popular tools for characterizing the mechanical properties of hydrogels is the static tensile test which provides information about the hydrogels’ tensile strength and the possibility of their elongation [39]. Tensile strength may be defined as the maximum stress that a material is able to withstand before its breakage [40]. 

Based on the results presented in Figure 11, it may be observed that the highest tensile strength—i.e., 0.112 MPa—was reported for unmodified hydrogel. Next, as the amount of the licorice root extract increased, the hydrogel tensile strength decreased, wherein the lowest value of this parameter—i.e., 0.072 MPa—was calculated for the sample containing 5 mL of the plant extract and 1 mL of nanosilver suspension (sample 5/1 nanoAg). The decrease in the value of the tensile strength results from the introduction into the material of additional modifying substances while simultaneously maintaining the same amount of crosslinking agent. The introduction of the plant extract and nanosilver suspension results in the dilution of the reaction mixture, while the use of the same amount of the crosslinker may result in the preparation of the material with a lower crosslinking density. However, it should be emphasized that in the case of the percentage elongation, which indicates the hydrogels’ elasticity, such changes in its values were not so visible. The elasticity of modified hydrogels compared to the elasticity of unmodified materials decreased slightly. The sample containing the highest amounts of the modifying substances—sample 5/1 nanoAg—is characterized by a percentage elongation of approximately 24.5%, which in the case of the application of such a material as dressing, is beneficial and consistent with previously presented research [41,42,43].

### 2.8. In Vitro Biological Analysis of Hydrogels via MTT Reduction Assay

**Figure 12:**
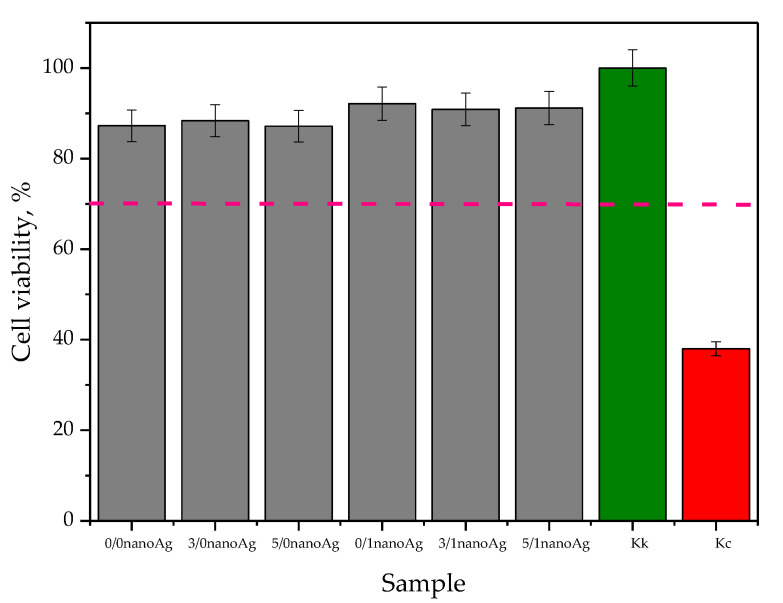
Results of in vitro cytotoxicity analysis of hydrogels via the MTT reduction assay.

In vitro cytotoxicity analysis was performed in line with EN ISO 10993-5:2009 standard [44]. Results of the MTT assay performed using L929 murine fibroblasts are presented in Figure 12, wherein the results of the statistical analysis are shown in Table 5. The study was performed in triplication, wherein the results are presented as average values with corresponding standard deviations (SD, given as error bars).

In accordance with the guidelines of the previously indicated standard, the material is defined as non-cytotoxic in the case when the viability of the selected cell line is incubated for 24 h in its presence above 70% (this cell viability has been marked in Figure 12 via the pink dotted line). Thus, in the case of all tested materials, this requirement was met, which confirms the lack of cytotoxic activity of developed hydrogels against the L929 murine fibroblasts. As it was demonstrated in Figure 12, in the case of samples containing silver nanoparticles, the cell viability slightly increased. This effect may be attributed to the antibacterial activity of nanosilver [45,46]. Developed materials that are applicable for biomedical uses were obtained in strictly controlled conditions, ensuring the highest possible sterility. However, during the synthesis, transport, or investigations, their slight contamination will occur; then, the presence of silver nanoparticles showing antibacterial properties may result in a slight increase in cell survival. This, in turn, constitutes an additional advantage of developed materials.

## 3. Materials and Methods

### 3.1. Materials

Chitosan (high molecular weight, deacetylation degree 75–85%), gelatin (obtained from porcine skin, Type A, gel strength 300), 2-hydroxy-2-methylpropiophenone (photoinitiator, d = 1.077 g/mL, 97%), diacrylate poly(ethylene glycol (crosslinking agent, d = 1.120 g/mL, average molecular weight Mn = 700 g/mol), and polyvinylpyrrolidone (average molecular weight 10,000 g/mol) were bought in Merck (Darmstadt, Germany). Silver nitrate (99.9%, pure p.a.) and sodium borohydride (NaBH_4_, 98%, pure p.a.) were purchased from Avantor Performance Materials Poland S.A. (Gliwice, Poland). *Glycyrrhiza glabra* (Licorice) root was bought in Natur-Sklep (Wrocław, Poland).

### 3.2. Preparation of Glycyrrhiza glabra (Licorice) Root Extract

**Figure 13:**
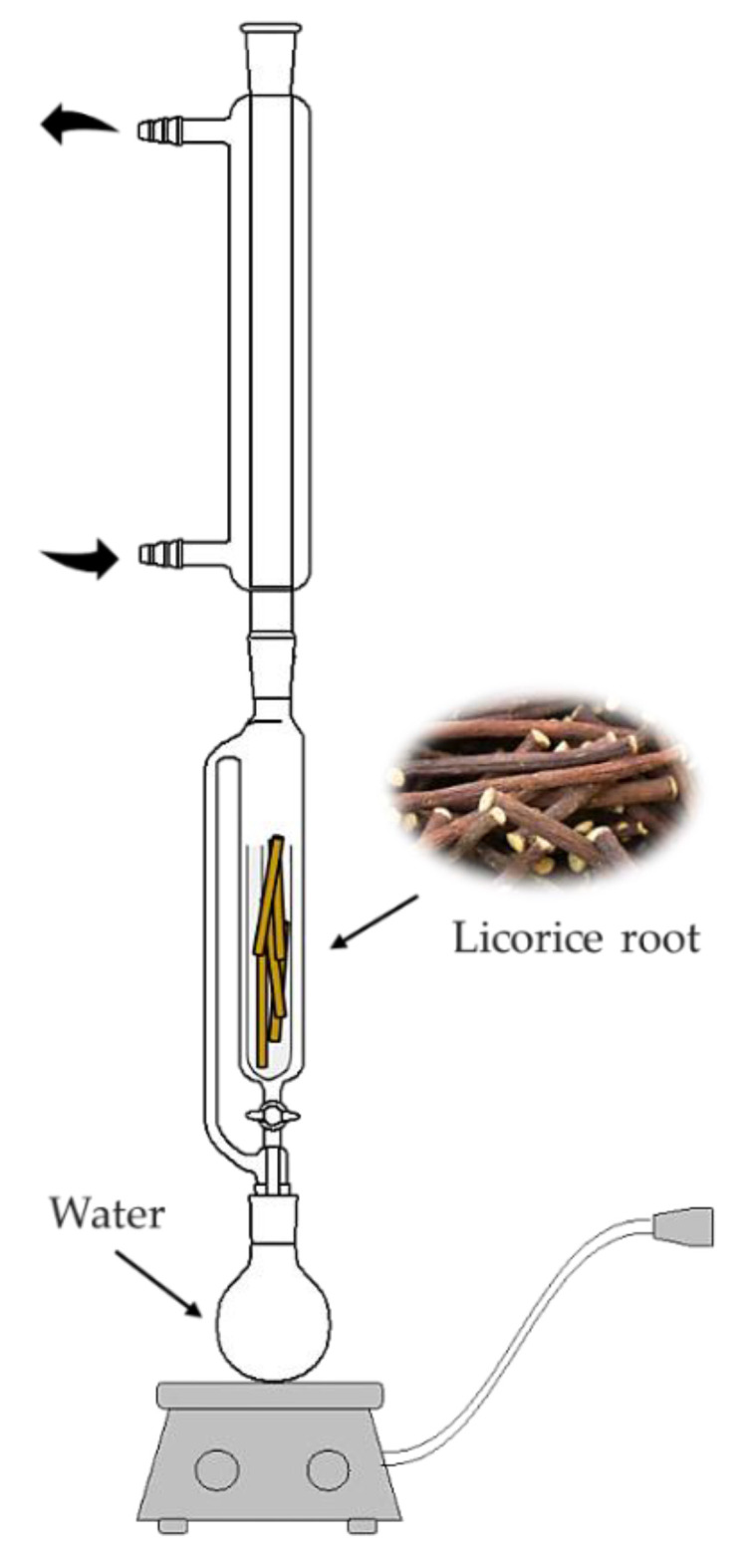
Licorice root extraction scheme.

In order to obtain bioactive components of licorice, the solid–liquid extraction using the Soxhlet extractor was performed. This is the first-choice type of extraction in the case of isolating organic compounds from plant materials. The scheme of this process is presented below in Figure 13.

The procedure of the extraction was as follows: firstly, the licorice root was placed in a thimble while the distilled water was placed in the round bottom flask. Then, the solvent was heated to the boiling point. Such a process was performed for 4 h while maintaining a mild boiling state. Obtained aqueous extract of licorice root was subsequently used as a modifying agent of hydrogels.

### 3.3. Synthesis of Silver Nanoparticles via the Chemical Reduction Process

Silver nanoparticles were prepared via chemical reduction in which a silver nitrate was used as a source of silver while sodium borohydride was used as a reducing agent. Firstly, 0.039 g AgNO_3_ (so 250 ppm Ag) was dissolved in a 3% aqueous PVP solution (mixture I). Next, a solution of NaBH_4_ in 3% PVP solution was prepared and introduced dropwise to mixture I. Such process was performed at constant stirring and at ambient temperature. After dropping, obtained mixture was maintained at constant stirring for 15 min. Next, it was centrifuged (13,000 rpm) for 20 min. The supernatant was decanted, wherein the residue was suspended in distilled water.

### 3.4. Characterization of Silver Nanoparticle Suspension

#### 3.4.1. The Particle Size Analysis via DLS Technique

The size of the particles obtained via the chemical reduction was verified using dynamic light scattering (DLS technique). For this purpose, a Zetasizer Nano ZS Malvern apparatus (Malvern Panalytical Ltd., Malvern, UK) was employed, wherein the measurements were performed at ambient temperature.

#### 3.4.2. Analysis of the Optical Properties of Nanosilver Suspension

Suspension of silver nanoparticles was also subjected to UV–Vis spectroscopy. The study aimed to determine the ability of nanoparticles to absorb light within the UV–Vis range. The analysis was conducted using a ThermoScientific Evolution 220 UV–Vis spectrometer (Thermo Fisher Scientific, Waltham, MA, USA) at room temperature.

### 3.5. Synthesis of Hydrogel Polymers via the Photopolymerization Process

In order to prepare hydrogel materials, the UV-induced photopolymerization process was employed. This method allows for obtaining hydrogels in a quick, waste-free, and low energy-demand manner. As a source of UV radiation, an EMITA VP-60 lamp (power 180 W, λ = 320 nm) was applied. Firstly, a 3% chitosan solution in 0.05% acetic acid solution and 2% gelatin solution was prepared. Next, adequate amounts of these solutions were mixed with adequate amounts of Glycyrrhiza glabra (licorice) root extract, nanosilver suspension, crosslinking agent, and photoinitiator. The mixtures obtained were thoroughly mixed, poured into the Petri dishes, and treated with UV radiation for 120 s. Detailed compositions of all prepared hydrogels are given below in Table 6.

After the synthesis, hydrogels were dried at 37 °C for 24 h and investigated to characterize their physicochemical and biological properties. The main attention was focused on determining the impact of the modifiers—licorice root extract and nanosilver suspension—on hydrogels’ properties. Moreover, the discussion over the results of performed experiments also included their evaluation in terms of their application as dressing materials.

### 3.6. Assessment of the Swelling Properties of Hydrogels

High swelling properties are one of the most characteristic features of hydrogels. Swelling ability of these materials is particularly important in terms of their potential use as dressings with wound exudate sorption function. Thus the hydrogels’ swelling capacity was verified in the artificial saliva, SBF, Ringer liquid, and distilled water. The procedure was as follows: dry hydrogel samples (with a diameter of 2 cm) were firstly accurately weighed and then placed in tested liquids (50 mL) for 1 h. Next, the materials were separated from the liquids, the excess liquid (unbound with the sample) was removed via the paper towel, and samples were weighed again. Subsequently, the hydrogels were placed again in the same liquids, and the procedure was repeated after 24 h and 72 h. The swelling ability of hydrogels was defined in each tested liquid and after each swelling period via the swelling ratio (*Q*) calculated by means of the following Equation (1):

(1) $$Q=\frac{m_{s}-m_{d}}{m_{d}}\times 100\%$$

where: *Q*—swelling ratio, %; $m_{s}$—weight of hydrogel after swelling for a specific time period (i.e., after 1 h, 24 h, or 72 h), g; $m_{d}$—weight of dry sample (before swelling), g.

The swelling studies were performed at ambient temperature and in triplicates for each sample.

### 3.7. Analysis of the Influence of Hydrogels on Simulated Physiological Fluids (Incubation Studies)

The incubation studies consisted of introduction of dry hydrogel samples (weighing approximately 1.0 g and with a diameter of 2.0 cm) into selected liquids for 12 days and measurement every two days of the pH and the temperature of the incubation medium. In terms of the potential application of tested materials for biomedical purposes (as dressing materials), the following liquids were selected for incubation: artificial saliva solution, Ringer liquid (infusion liquid used to restore the body’s water-electrolyte balance), simulated body fluid (SBF, isotonic to human blood plasma) and distilled water (as a reference liquid). The study aimed to verify whether hydrogel affects the parameters of the liquids. The measurements were performed using the multifunctional ELMETRON CX-701 (Elmetron, Zabrze, Poland) meter. In order to simulate conditions occurring in the human body to a greater extent, the incubation was performed at 37 °C. The study was conducted in triplicates for each sample.

### 3.8. Evaluation of the Impact of Hydrogels’ Incubation on Their Chemical Structure via FT-IR Spectroscopy

Hydrogel samples, after incubation in simulated physiological liquids, were subjected to FT-IR spectroscopy to verify potential changes in their structures resulting from the incubation. Such changes could indicate, e.g., the degradation of the hydrogels. The spectroscopic analysis was carried out using the Nicolet iS5 Thermo Scientific (Thermo Fischer Scientific, Waltham, MA, USA) spectrometer, wherein the spectra were recorded within the range 4500–500 cm^−1^ and at a resolution of 4.0 cm^−1^.

### 3.9. Analysis of the Surface Morphology Using SEM Technique

The next step in the research involved characterization of hydrogels’ surface morphology. For this purpose, dry hydrogel samples (with dimensions 1 cm × 1 cm) were sputtered with gold and subjected to the analysis using scanning electron microscopy, wherein the study was conducted by means of a Jeol 5510 LV (Jeol Ltd., Tokyo, Japan) microscope. The imaging was conducted at ambient temperature.

### 3.10. Studies on the Wettability of Hydrogels Supported by Determining the Surface Free Energy

The hydrogels were also subjected to the analysis of their wettability. For this purpose, hydrogel samples were treated with a drop of double distilled water dispensed from a syringe. The procedure was conducted with simultaneous recording of the behavior of the drop of the wetting liquid during its first contact with the tested material. Therefore, as a result of the study, the wetting angle for each sample, as well as the images showing the placement of the drop on its surface, were obtained. The measurements were performed at ambient temperature using the Drop Shape Analyzer Kruss DSA100 M measuring instrument (Gmbh, Hamburg, Germany). The whole procedure of the analysis was described in more detail in our previous paper [47]. Importantly, the analysis also enabled the calculation of the surface free energy via the Owens-Wendt method [48].

### 3.11. Characteristics of the Mechanical Properties of Hydrogels

Hydrogels were also subjected to the analysis of their mechanical properties, including determining their percentage elongation and tensile strength. The study was performed according to the ISO 37 type 2 and ISO 527-2 type 5A standards, wherein the universal testing machine (Shimadzu, Kyoto, Japan) was applied for the measurements. Firstly, after the synthesis, the paddle-shaped hydrogel samples were prepared using the ZCP020 manual blanking press, and they were next dried under pressure (to keep the shape) at 37 °C for 24 h. Then, the measurements were performed, during which hydrogel samples were placed between the jaws of the machine. During the analysis, the jaws moved apart, proceeding with simultaneous sample stretching. The procedure was carried on until the sample breakage. The measurements were performed at ambient temperature. Such an analysis allowed to determine the hydrogels’ tensile strength ($R_{m}$) using Equation (2) and the percentage elongation (A) using Equation (3). Both equations are presented below:

(2) $$R_{m}=\frac{Fm}{S_{0}}$$

(3) $$A=\frac{\left(l_{u}-l_{0}\right)}{l_{0}}\times 100\%$$

where: $F_{m}$—maximum hydrogel’s strength; $S_{0}$—cross-sectional area of sample in its initial state (before the analysis); $I_{u}$—measuring length after sample breakage; $I_{0}$—measuring length of sample in its initial state (before the analysis).

### 3.12. In Vitro MTT Reduction Assay Using L929 Murine Fibroblasts

In addition to characterizing the physicochemical properties of hydrogels, the key aspect was to verify their cytotoxicity towards selected cell lines. For this purpose, in vitro MTT reduction assay was employed, wherein, as tested cell lines, L929 murine fibroblasts were selected. Conducting this type of preliminary biological investigation provides information on whether the chosen synthesis methodology, as well as the composition of the developed materials, leads to the preparation of materials that could be considered for more advanced biological experiments. When such an assay indicates cytotoxicity of the hydrogels, then the synthesis methodology or the amounts of individual reagents applied during the synthesis needs to be modified. The principle of MTT reduction assay is to check the cell viability by determining their metabolic activity. For this purpose, the MTT reagent (i.e., 3-(4,5-dimethylthiazol-2-yl)-2,5-diphenyltetrazolium bromide; tetrazolium salt) is added to the medium with tested cell lines (here: L929 murine fibroblasts). Metabolically active cells secrete into the culture medium mitochondrial dehydrogenase, and this enzyme converts the MTT reagent into formazan. The blue crystals of formazan are next dissolved in the organic solvent (e.g., in dimethyl sulfoxide (DMSO)), and obtained solution may be next analyzed via UV–Vis spectroscopy. The absorbance of the solution corresponds to its concentration and, thus, to the amount of the enzyme present in the tested medium. In turn, the amount of the enzyme provides information on the number of viable cells. The procedure of MTT reduction assay, as well as L929 murine fibroblast culture, were described more precisely in our previous publication [49].

### 3.13. Statistical Analysis

The results of the research were subjected to statistical analysis wherein the statistical importance was determined by means of the two-way analysis of variance (ANOVA) (*α* = 5%). The calculations were performed in the case of the results of the mechanical studies, contact angle measurements, and biological studies (MTT reduction assay). The statistical analysis was carried out to verify the importance of the modifying factors—i.e., licorice root extract and nanosilver suspension. All experiments were performed in triplicates, and their results are provided, including the average value and the standard deviation (SD).

## 4. Conclusions

- All developed materials showed swelling ability. The modification of hydrogels with licorice root extract did not significantly affect this property. However, the incorporation of hydrogels with nanosilver resulted in an increase in this ability, which was probably caused by the interactions between aqueous nanosilver suspension and the liquid penetrating the polymer matrix.
- Incubation studies in simulated physiological liquids supported by the analysis of the structure of incubated hydrogels via FT-IR spectroscopy excluded the degradation of tested materials in these environments. The only slight changes (by a maximum of one pH unit) in the pH of incubation media reported in the course of the incubation probably resulted from the interactions between the components of the polymer matrix and the incubation media.
- Based on the SEM imaging, it was reported that licorice root extract may fill the outer cavities of the hydrogels, which results in the smoothing of its surface.
- The surface wettability of modified hydrogels indicated their hydrophilicity. The wetting angles of all tested samples were lower than 90°. The lowest wetting angles and the highest surface free energies were determined for hydrogels modified with the highest amounts of the additives (i.e., 5 mL of licorice root extract and 1 mL of nanosilver suspension).
- The introduction of the modifying agents into the hydrogels reduced their tensile strength from 0.112 MPa (for unmodified hydrogels) to 0.072 MPa (for the materials with the highest amounts of additives). However, the changes in the percentage elongations between unmodified materials and modified ones were not as significant. The hydrogel containing the highest amounts of additives showed approximately 24% elongation.
- In vitro biological analysis with L929 murine fibroblasts excluded the cytotoxic activity of the hydrogels; the viability of tested cells was within the range of 87.0–92.5%. Nanosilver present in hydrogel matrices positively affected this property and increased the cell viability.
- The results of the physicochemical analysis confirming the stability of the tested hydrogels in simulated physiological liquids, the possibility of their modification, and the lack of cytotoxic activity proved the correctness of the synthesis methodology applied. Moreover, the sorption properties of hydrogels indicated the possibility of absorbing wound exudate by these materials, while their hydrophilic surfaces demonstrated that they constitute a suitable substrate for cell adhesion and proliferation. Furthermore, their elasticity of approximately 30% flexibility indicated the possibility of their easy application. Thus, in summary, the above-mentioned features confirm the possibility of the use of developed hydrogels as dressing materials supporting regenerative processes.

## Figures and Tables

**Table 1:** Results of hydrogels’ wettability analysis.

Sample Name	Total Surface Free Energy, mJ/m^2^	Contact Angle, °	Image of Hydrogel during Its First Contact with Water
0/0 nanoAg	55.22	42.85 ± 0.68	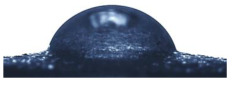
3/0 nanoAg	60.58	35.15 ± 1.15	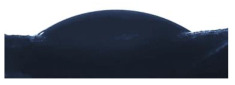
5/0 nanoAg	67.67	29.17 ± 0.93	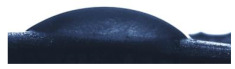
0/1 nanoAg	55.88	41.45 ± 0.97	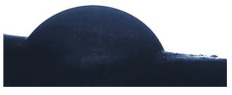
3/1 nanoAg	62.28	33.85 ± 1.02	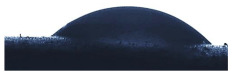
5/1 nanoAg	72.08	26.58 ± 1.18	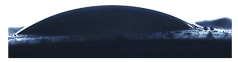

**Table 2:** Results of the statistical analysis of contact angle measurements performed via the two-way analysis of variance (ANOVA) (*p* indicates the statistical significance).

Analysis of Variance	*p*
Source of Variation	
Licorice root extract	0.02275
Nanosilver suspension	0.002
Interaction	0.99443

**Table 3:** Results of the statistical analysis of hydrogels’ tensile strength measurements performed via the two-way analysis of variance (ANOVA) (*p* indicates the statistical significance).

Analysis of Variance	*p*
Source of Variation	
Licorice root extract	0.01991
Nanosilver suspension	3.8525 × 10^−4^
Interaction	0.01991

**Table 4:** Results of the statistical analysis of hydrogels’ percentage elongation measurements performed via the two-way analysis of variance (ANOVA) (*p* indicates the statistical significance).

Analysis of Variance	*p*
Source of Variation	
Licorice root extract	0.02092
Nanosilver suspension	0.00634
Interaction	0.02094

**Table 5:** Results of the statistical analysis of MTT reduction assay conducted via the two-way analysis of variance (ANOVA) (*p* indicates the statistical significance).

Analysis of Variance	*p*
Source of Variation	
Licorice root extract	0.03264
Nanosilver suspension	5.32907 × 10^−4^
Interaction	0.03264

**Table 6:** Compositions of prepared hydrogels.

No.	3% Chitosan Solution, mL	2% Gelatin Solution, mL	Crosslinking Agent, mL	Photoinitiator, mL	Licorice Root Extract, mL	Nanosilver Suspension, mL	Sample
1.	30	20	8	0.25	-	-	0/0 nanoAg
2.					3	-	3/0 nanoAg
3.					5	-	5/0 nanoAg
4.					-	1	0/1 nanoAg
5.					3	1	3/1 nanoAg
6.					5	1	5/1 nanoAg

## Data Availability

Data sharing is not applicable to this article.

## Abbreviations

FT-IR spectroscopy, Fourier transform infrared spectroscopy; MTT, 3-(4,5-dimethylthiazol-2-yl)-2,5-diphenyltetrazolium bromide; HRSV, human respiratory syncytial virus; UV, ultraviolet; DLS, dynamic light scattering; UV–Vis, ultraviolet–visible, SD, standard deviation; SEM, scanning electron microscopy; EN ISO, English International Organization for Standardization; PVP, polyvinylpyrrolidone; SBF, simulated body fluid; DMSO, dimethyl sulfoxide.
